# What is the outcome of the open IPOM versus sublay technique in the treatment of larger incisional hernias?: A propensity score-matched comparison of 9091 patients from the Herniamed Registry

**DOI:** 10.1007/s10029-020-02143-4

**Published:** 2020-02-25

**Authors:** F. Köckerling, B. Lammers, D. Weyhe, W. Reinpold, K. Zarras, D. Adolf, H. Riediger, C. M. Krüger

**Affiliations:** 1 Department of Surgery and Center for Minimally Invasive Surgery, Academic Teaching Hospital of Charité Medical School, Vivantes Hospital, Neue Bergstrasse 6, 13585 Berlin, Germany; 2 Department of Surgery I – Section Coloproctology and Hernia Surgery, Lukas Hospital, Preussenstr. 84, 41464 Neuss, Germany; 3 University Clinic for Visceral Surgery, Pius Hospital Oldenburg, Georgstraße 12, 26121 Oldenburg, Germany; 4 Wilhelmsburger Hospital Groß-Sand, Groß-Sand 3, 21107 Hamburg, Germany; 5 Marien Hospital Düsseldorf, Rochusstraße 2, 40479 Düsseldorf, Germany; 6 StatConsult GmbH, Halberstädter Strasse 40 a, 39112 Magdeburg, Germany; 7 Vivantes Humboldt Hospital, Am Nordgraben 2, 13509 Berlin, Germany; 8 Immanuel Hospital Rüdersdorf, Seebad 82/83, 155562 Rüdersdorf, Germany

**Keywords:** Incisional hernia, Open IPOM, Sublay, Chronic pain, Postoperative complications, Recurrence

## Abstract

**Introduction:**

In an Expert Consensus guided by systematic review, the panel agreed that for open elective incisional hernia repair, sublay mesh location is preferred, but open intraperitoneal onlay mesh (IPOM) may be useful in certain settings. This analysis of data from the Herniamed Registry aimed to compare the outcomes of open IPOM and sublay technique.

**Methods:**

Propensity score matching of 9091 patients with elective incisional hernia repair and with defect width ≥ 4 cm was performed. The following matching variables were selected: age, gender, risk factors, ASA score, preoperative pain, defect size, and defect localization.

**Results:**

For the 1977 patients with open IPOM repair and 7114 patients with sublay repair, *n* = 1938 (98%) pairs were formed. No differences were seen between the two groups with regard to the intraoperative, postoperative and general complications, complication-related reoperations and recurrences. But significant disadvantages were identified for the open IPOM repair in respect of pain on exertion (17.1% vs. 13.7%; *p* = 0.007), pain at rest (10.4% vs. 8.3%; *p* = 0.040) and chronic pain requiring treatment (8.8% vs. 5.8%; *p* < 0.001), in addition to rates of 3.8%, 1.1% and 1.1%, respectively, occurring in both matched patients. No relationship with tacker mesh fixation was identified. There are only very few reports in the literature with comparable findings.

**Conclusion:**

Compared with sublay repair, open IPOM repair appears to pose a higher risk of chronic pain. This finding concords with the Expert Consensus recommending that incisional hernia should preferably be repaired using the sublay technique.

## Introduction

Two recently published systematic reviews and meta-analyses once again unequivocally demonstrated that the use of mesh procedures for repair of ventral and incisional hernias produces better results than suture techniques [1, 2]. This was also confirmed by the five-year findings of the Danish Hernia Database [3]. Therefore, with grade A level of evidence, an Expert Consensus recommends the use of a mesh for ventral and incisional hernias ≥ 2 cm [4]. However, which mesh procedure should best be used for which patient continues to be the subject of debate due to the absence of adequate data [4]. Based on meta-analyses [5–8], registry data [9] and guidelines [10–16], laparoscopic IPOM can be recommended for repair of incisional hernia defects up to 8–10 cm. Compared with open mesh procedures, laparoscopic IPOM is associated with fewer surgical site occurrences and complication-related reoperations [5–16]. But for a defect size greater than 8–10 cm, the recurrence rate rises sharply following laparoscopic IPOM [10–16]. For the open mesh procedures for treatment of ventral and incisional hernias, systematic reviews and meta-analyses show the best outcomes for the sublay repair [17–20]. In the Expert Consensus guided by systematic review, the panel agreed that for open elective incisional hernia repair, sublay mesh location is preferred, but open intraperitoneal onlay mesh (IPOM) and onlay mesh may be useful in certain settings [4]. In a propensity score-matched comparison of data from the Herniamed Registry small and lateral placed incisional hernias can be safely managed with an onlay repair [21]. A literature review of open IPOM for incisional hernia repair found high variance of 3.3–72.0% with a mean value of 20.4% for the postoperative complication rates and of 0–61% with a mean value of 12.6% for the recurrence rates [22].

The following analysis of data from the Herniamed Registry now compares the perioperative and one-year follow-up outcome for open IPOM versus sublay technique for elective incisional hernia repair with a defect size of ≥ 4 cm. Propensity score matching was applied to obtain comparable patient collectives [23].

## Methods

As of February 1, 2019, 712 hospitals and surgeons in independent practice in Germany, Austria and Switzerland were taking part in the internet-based Herniamed Hernia Registry [24, 25]. The participating hospitals and self-employed surgeons enter data prospectively into the Herniamed Registry on the routine operations performed by them for patients with abdominal wall hernias, subject to the patient having signed a consent form to that effect.

As part of the information provided to patients regarding participation in the Herniamed Registry and signing the informed consent declaration, all patients were informed that the treating hospital or surgeon would like to be informed about any problem occurring after the operation and that the patient had the opportunity to attend for clinical examination [9, 26].

All postoperative complications occurring within 30 days should be identified and documented. Therefore, the questionnaire sent to the general practitioner and patient at one-year follow-up once again enquires about any postoperative complications [9, 26]. Furthermore, in the one-year follow-up questionnaire, both the patient and general practitioner are asked about any recurrence, bulging, pain at rest, pain on exertion, and chronic pain requiring treatment [9, 26]. If recurrence or chronic pain was reported by the general practitioner or patient, patients could be requested to attend clinical examination or radiologic tests [9, 26].

One publication has provided impressive evidence of the role of patient-reported outcomes for both recurrence and chronic pain [27].

**Fig. 1:**
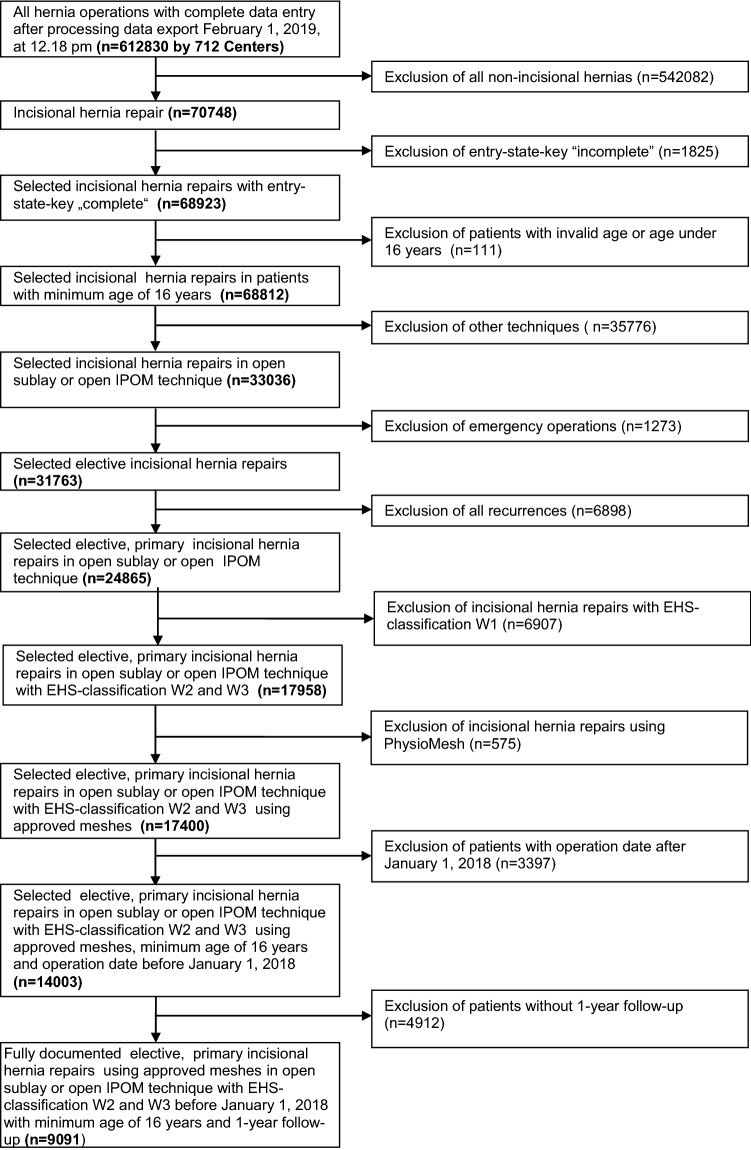
Flowchart of patient inclusion

The following analysis compares the prospective data of all patients with primary incisional hernia repair and a defect size of ≥ 4 cm [EHS defect width II (≥ 4–10 cm), III (> 10 cm)] [28] operated on with either open IPOM or open sublay technique. Only patients at least 16 years old, who had undergone elective incisional hernia repair and with one-year follow-up, were included (Fig. 1). In total, 9091 patients were included between September 1, 2009 and January 1, 2018 (Fig. 1).

The demographic and patient-related parameters include age (years), gender, American Society of Anesthesiologists (ASA) score I, II, III–IV, BMI (kg/m^2^), and risk factors like chronic obstructive pulmonary disease (COPD), diabetes mellitus, aortic aneurysms, corticoid medication, immunosuppression, coagulopathy, smoking, antiplatelet medication, anticoagulant therapy, and preoperative pain. Hernia-related variables influencing the outcome included the hernia defect size according to the European Hernia Society (EHS) classification (W2 ≥ 4–10 cm, and W3 > 10 cm) [28] and hernia localization (medial, lateral, and combined) [28]. Hernia width was recorded during surgery based on intraoperative measurements [9, 21, 26]. The dependent variables were intraoperative, postoperative and general complication rates, complication-related reoperation rate, recurrence rate and rates of pain at rest, pain on exertion, and chronic pain requiring treatment [9, 21, 26].

All analyses were performed with the software SAS 9.4 (SAS Institute Inc., Cary, NC, USA) and intentionally calculated to a full significance level of 5%, that is they were not corrected for multiple testing and each *p* value ≤ 0.05 represents a significant result [9, 21, 26]. The individual outcome and influencing variables (risk factors and complications) were summarized as global variables. A general, intra- or postoperative complication or risk factor was deemed to be present if at least one single item was applied [9, 21, 26].

Propensity score matching is a suitable statistical method for formation of comparison groups from a very heterogeneous patient population. Persons with similar characteristics were assigned to the comparison groups and then compared with regard to the outcome variables. The propensity scores were calculated using a logistic regression model with selected matching variables. The following matching variables were selected: age in years, BMI (kg/m^2^), gender (male/female as a fixed variable), risk factors (yes/no), ASA score (I, II, and III–IV), preoperative pain (yes, no, and unknown), defect size (W2 ≥ 4–10 cm, W3 > 10 cm), drainage (yes/no), and EHS classification (medial, lateral, and combined) [9, 21].

The robust greedy algorithm was used for matching applying a caliper of 0.1 standard deviation [9, 21]. Unadjusted analyses were performed before matching for analysis of the operation techniques with regard to the matching parameters. This helped to obtain a description of the patient collective before matching. The asymptotic Chi-square test was used for categorical parameters and the robust t test (Satterthwaite) for continuous parameters [9, 21]. To assess the balance of the single matching parameters between comparison groups after matching, standardized differences were estimated. As a rule of thumb, a good balance between the groups and, thus, comparability is assured by a standardized difference of less than 10% (< 0.1) [9, 21].

McNemar’s test was performed to analyze the influence of the operation techniques on the outcome parameters (general, intra- and postoperative complications, complication-related reoperations, pain at rest, pain on exertion, chronic pain requiring treatment, and recurrence at one-year follow-up) in the matched samples [9, 21].

Furthermore, odds ratio estimates (adjusted for matched samples) and their corresponding 95% confidence intervals are given [9, 21].

## Results

Following patient selection, 9091 patients were finally included in propensity score matching analysis to create homogeneous comparison groups (Fig. 1). Of these patients, 1977 were operated on with the open IPOM and 7114 patients with the open sublay technique. No significant difference was found for the continuous variables age (mean ± SD: open IPOM 64.5 ± 12.5 years vs. open sublay 64.5 ± 12.3 years; *p* = 0.903) and body mass index (BMI) (mean ± SD: open IPOM 29.5 ± 5.8 kg/m^2^ vs. open sublay 29.3 ± 5.8; *p* = 0.141). For the categorical influence factors, significant differences were identified between these two surgical techniques with regard to the EHS width classification, EHS defect classification, drainage and preoperative pain (Table 1).

**Table 1:** Patient and procedure related categorical influencing factors on the outcome

	Procedure				*p*
	Open IPOM		Open sublay		
	*n*	%	*n*	%	
Gender					
Male	1038	52.50	3712	52.18	0.798
Female	939	47.50	3402	47.82	
ASA score					
I	155	7.84	559	7.86	0.571
II	1088	55.03	4004	56.28	
III/IV	734	37.13	2551	35.86	
Defect size					
W2	1283	64.90	5111	71.84	< 0.001
W3	694	35.10	2003	28.16	
EHS classification					
Combined	254	12.85	557	7.83	< 0.001
Lateral	317	16.03	998	14.03	
Medial	1406	71.12	5559	78.14	
Preoperative pain					
Yes	1044	52.81	3992	56.11	0.031
No	760	38.44	2530	35.56	
Unknown	173	8.75	592	8.32	
Drainage					
Yes	1371	69.35	6429	90.37	< 0.001
No	606	30.65	685	9.63	
Risk factors					
Total					
Yes	892	45.12	3232	45.43	0.805
No	1085	54.88	3882	54.57	
COPD					
Yes	252	12.75	828	11.64	0.178
No	1725	87.25	6286	88.36	
Diabetes					
Yes	309	15.63	1010	14.20	0.110
No	1668	84.37	6104	85.80	
Aortic aneurysm					
Yes	40	2.02	176	2.47	0.244
No	1937	97.98	6938	97.53	
Immunosuppression					
Yes	45	2.28	148	2.08	0.593
No	1932	97.72	6966	97.92	
Corticoid therapy					
Yes	41	2.07	126	1.77	0.375
No	1936	97.93	6988	98.23	
Smoking					
Yes	252	12.75	963	13.54	0.361
No	1725	87.25	6151	86.46	
Coagulopathy					
Yes	51	2.58	175	2.46	0.762
No	1926	97.42	6939	97.54	
ASS/Plavix Antiplatelet medication					
Yes	241	12.19	1021	14.35	0.014
No	1736	87.81	6093	85.65	
Anticoagulation therapy					
Yes	74	3.74	235	3.30	0.340
No	1903	96.26	6879	96.70	

For example, significantly more patients who had undergone open IPOM repair had defect size W3 (> 10 cm) and lateral and combined defect localization than those operated on with open sublay technique. Patients with sublay repair already had preoperative pain more often. For the open sublay technique, drains were also used significantly more often.

**Fig. 2:**
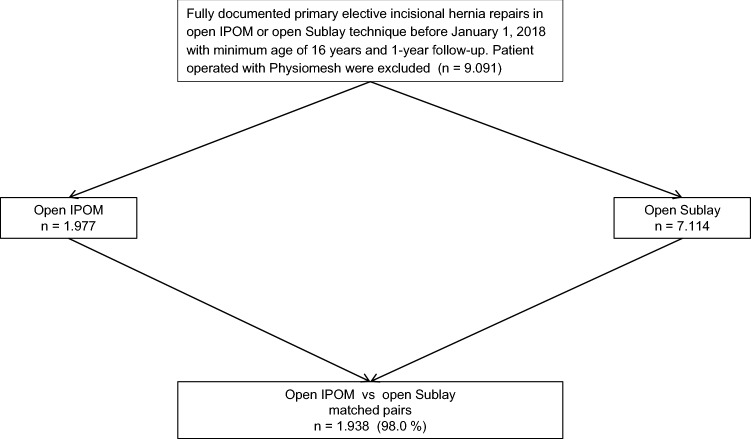
Flowchart of patient matching

The greedy algorithm was used for propensity score matching of the 1977 patients with open IPOM repair to the 7114 patients with open sublay repair, while applying a caliper of 0.1 standard deviation. Matching was performed for *n* = 1938 (98%) of patients (Fig. 2).

**Fig. 3:**
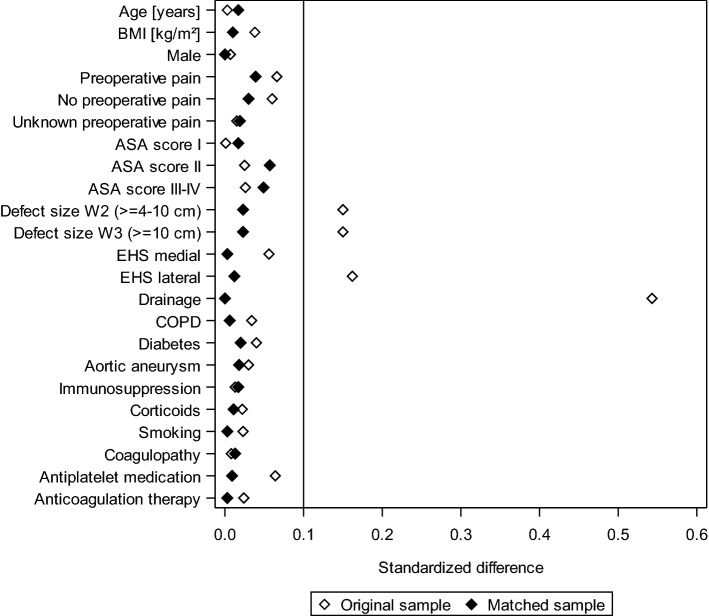
Standardized differences between the matching variables both before (original sample) and after matching (matched sample)

**Fig. 4:**
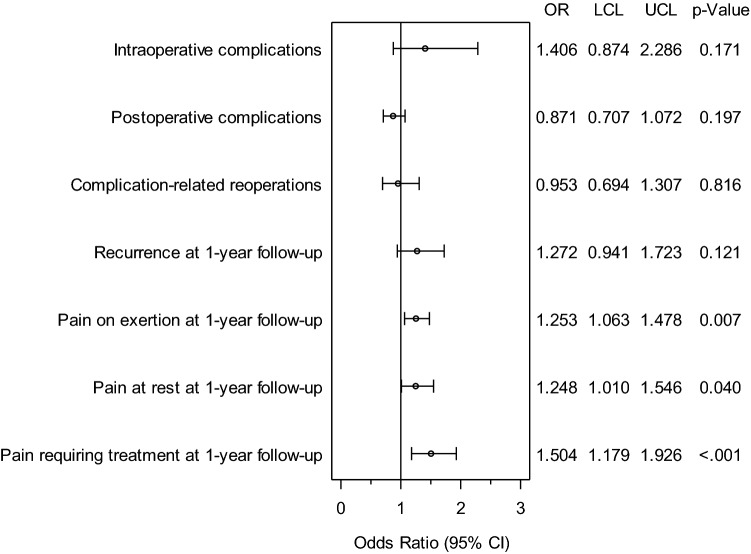
Results of matched pairs analysis of incisional hernia repair with open IPOM versus open Sublay procedures

The standardized differences between the matching variables both before (original sample) and after matching (matched sample) are shown below (Fig. 3). The difference after matching is less than 10% for all matching variables, indicating a good balance for the variables included in the model (Fig. 3). The results of matched pair analysis of both surgical techniques are summarized for the various outcome parameters in Table 2 and Fig. 4. Systematic differences between the surgical techniques were identified on follow-up only for the pain rates. For pain on exertion, a significant difference was found in favor of the patients operated on with the open sublay procedure (13.7% vs. 17.1%; *p* = 0.007) in addition to a rate of 3.8% pairs with pain occurring in both matched patients. Likewise, for pain at rest (8.3% vs. 10.4%; *p* = 0.040; plus concordant 1.1% cases with pain) and chronic pain requiring treatment (5.8% vs. 8.8%; *p* < 0.001; plus concordant 1.1% cases with pain), a difference was seen in favor of the open sublay technique. No systematic difference was found between the two surgical techniques for any of the other outcome variables.

**Table 2:** Results of matched pair analysis of incisional hernia repair in open IPOM versus open Sublay technique (*n* = 1.938 pairs)

	Disadvantages		*p* value	OR for matched samples		
	Open IPOM	Open sublay		OR		
Intraoperative complication	2.32	1.65	0.171	1.406	0.874	2.286
General complication	5.78	5.06	0.370	1.143	0.864	1.514
Postoperative complication	9.03	10.37	0.197	0.871	0.707	1.072
Complication-related reoperation	4.18	4.39	0.816	0.953	0.694	1.307
Recurrence on 1-year follow-up	5.31	4.18	0.121	1.272	0.941	1.723
Pain on exertion on 1-year follow-up	17.13	13.67	0.007	1.253	1.063	1.478
Pain in rest on 1-year follow-up	10.37	8.31	0.040	1.248	1.010	1.546
Pain requiring treatment on 1-year follow-up	8.77	5.83	< 0.001	1.504	1.179	1.926

### Additional analysis of mesh fixation

To investigate the reasons for the significantly higher pain rates associated with open IPOM, the influence of using tackers for mesh fixation was analyzed additionally.

For 760 of 1938 patients with open IPOM (39.2%) the mesh was fixed only with tackers or in combination with tackers. Compared with the patient group with no tacker mesh fixation, and only with suture fixation, no significant influence was identified on the pain rates at one-year follow-up (Table 3). As the Herniamed Registry does not contain further information on the type of suture mesh fixation (transfascial, single or running suture), a possibly negative influence of transfascial sutures on the chronic pain rates could not be analyzed.

**Table 3:** Influence of tacker mesh fixation on pain rates in 1-year follow-up following incisional hernia repair in open IPOM technique

	Tacker or combination with tacker				*p*
	Yes		No		
	*n*	%	*n*	%	
Pain on exertion on 1-year follow-up					
Yes	156	20.53	250	21.22	0.713
No	604	79.47	928	78.78	
Pain in rest on 1-year follow-up					
Yes	77	10.13	146	12.39	0.128
No	683	89.87	1032	87.61	
Pain requiring treatment on 1-year follow-up					
Yes	65	8.55	126	10.70	0.122
No	695	91.45	1052	89.30	

### Additional analysis of patients without follow-up

**Fig. 5:**
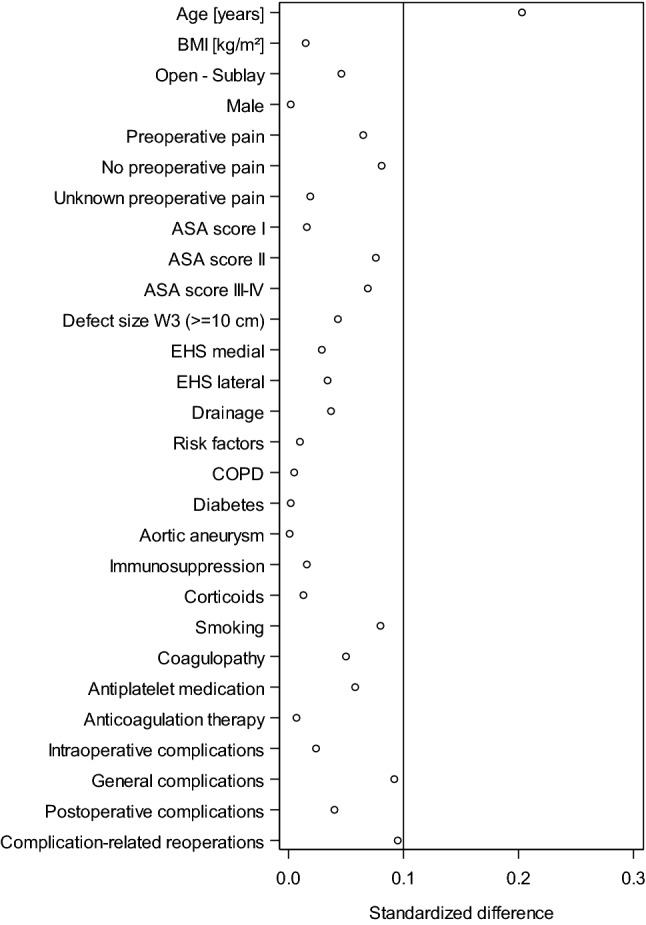
Standardized differences of the influencing factors and the perioperative outcomes between patient collectives with and without follow-up

To investigate whether there were relevant differences between the populations with and without one-year follow-up, standardized differences were calculated for all patient- and operation-related variables as well as perioperative outcome variables (Fig. 5). With the exception of age, for all other factors, the standardized difference was found to be below 0.1. Thus, there is no relevant bias in selection of patients due to availability of follow-up information.

## Discussion

Based on the propensity score matching analysis presented here for 1938 pairs, it can be demonstrated that open IPOM compared with sublay repair for incisional hernia repair has significant disadvantages. Pain on exertion, pain at rest and chronic pain requiring treatment occurred significantly more often after open IPOM compared with sublay technique. However, for the other outcome criteria, intraoperative, postoperative and general complications, complicated-related reoperations and recurrences, no systematic disadvantage was identified for the open IPOM compared with the sublay technique. Here, it must be noted that the open IPOM technique was used more often for larger defects (EHS width classification III (> 10 cm)) and lateral and combined defects. Patients with open IPOM repair also reported preoperative pain less often than patients with sublay repair. Hence, propensity score matching revealed that the comparative patient collectives were characterized by a higher proportion of incisional hernias with EHS width classification III (> 10 cm) and lateral and combined localization as well as a smaller proportion of patients with preoperative pain.

The use of tackers for mesh fixation in open IPOM was not found to significantly influence the chronic pain rates. Data of the possible influence of transfascial sutures on the chronic pain rates were not available.

In systematic reviews and meta-analyses of open surgical techniques for incisional hernia repair, the only details reported on the outcome relate to the perioperative complications and recurrences [17–19].

There are hardly any reports in the literature on chronic pain rates following incisional hernia repair. In a prospective study of 109 patients with elective incisional hernia repair with underlay composite mesh placement, chronic pain was defined as significant pain persisting after 3 months as assessed using a 10-point numeric scale (≥ 3 chronic pain, ≥ 7 severe pain) [29]. After a mean follow-up period of 24.6 months, 28% of the patients had chronic pain and 6.6% had severe pain. The authors concluded that chronic pain is not uncommon after intraperitoneal composite mesh placement for incisional hernia repair [29].

In a Cochrane review of open surgical procedures for incisional hernia repair, more postoperative pain was found in the intraperitoneal mesh group [30].

Hence, it must be assumed that there are essentially no differences in the perioperative outcome and recurrence rate following incisional hernia repair in IPOM technique compared with sublay repair but that a higher rate of chronic pain must be expected. This appears to be independent of the tacker in comparison to suture mesh fixation technique. Therefore, the open IPOM technique should only be used when sublay repair is not possible. As such, the findings presented here concord with the recommendation of the Expert Consensus, indicating that for open elective incisional hernia repair, sublay mesh location is preferred, but open intraperitoneal onlay mesh may be useful in certain settings [4].

Incorrect or missing data limit a registry [9, 21, 26, 31]. In the Herniamed Registry, all participating surgeons and chairmen of surgical departments sign a contract for the data correctness and completeness [9, 21, 26, 31]. As part of the certification process of hernia centers, data entry can be controlled by experts [9, 21, 26, 31]. Postoperative outcomes are once again reviewed at one-year follow-up [9, 21, 26, 31]. Since there are few reports on chronic pain after open IPOM repair of incisional hernias, comparison with the literature is accordingly very limited.

In summary, there is no systematic difference in the perioperative outcome or recurrence rate when comparing the open IPOM with the sublay technique for elective incisional hernia repair for large defects (EHS W2 ≥ 4–10 cm, EHS W3 > 10 cm). However, significantly higher rates of pain on exertion, pain at rest and chronic pain requiring treatment to the disadvantage of open IPOM repair were identified. No relationship with tacker mesh fixation was identified. Hence, the open IPOM technique should only be used when sublay repair is not possible.
